# Experiences of Social Relationships for Adults Living With Multiple Long‐Term Conditions: A Qualitative Interview Study

**DOI:** 10.1111/hex.70335

**Published:** 2025-09-10

**Authors:** Hilda Hounkpatin, Leanne Morrison, Al Richards, Miriam Santer

**Affiliations:** ^1^ Primary Care Research Centre, School of Primary Care, Population Sciences and Medical Education University of Southampton Southampton Hampshire UK

**Keywords:** loneliness, multiple long‐term conditions, qualitative, social isolation, social networks, social relationships

## Abstract

**Background:**

Social relationships are important for self‐management and outcomes of multiple long‐term conditions (MLTC). Previous research indicates MLTC negatively impacts social relationships and people living with MLTC do not feel adequately supported to manage their health. However, there is limited understanding of the processes and contextual factors that influence social relationships in the context of MLTC. This study explored experiences of social relationships for adults living with MLTC to improve understanding of their social relationship needs.

**Methods:**

Semi‐structured telephone interviews were conducted with 22 people living with MLTC in Southern England. Participants were recruited through charity networks and GP practices. Eligibility criteria were: individuals aged ≥ 45 years living with MLTC within the community. Participants were purposively sampled to include diverse characteristics in terms of age, gender, and residential area deprivation. Transcribed interview data were analysed using reflexive thematic analysis.

**Results:**

Four themes were developed. ‘There is no single route to meaningful social connection’ reflected how participants achieved, valued, and maintained meaning in relationships in diverse ways. ‘Change in sense of self’ captured the mental load, nostalgia, low mood and depressive symptoms experienced because of MLTC, which was reported to negatively impact their relationships with others. ‘A need to be seen and understood’ described how participants valued social relationships that enabled them to talk about their conditions with others who have shared experiences. Participants experienced a sense of abandonment when they felt unable to share their needs and experiences with family and close friends. Some participants experienced distress around the need to self‐manage their health, which was reinforced by limited health and care services. ‘Altered interaction’ reflected how MLTC required participants to find new ways to maintain social connection.

**Conclusion:**

Meaningful connection may be achieved through diverse social relationships, including acquaintances and support groups. Further efforts to connect people to these forms of relationships could improve self‐management of MLTC, though strategies may vary for adults with different characteristics and health needs. Interventions that target mental burden, nostalgia, low mood and depressive symptoms experienced because of MLTC could support appropriate and meaningful social connection. Further research is needed to test these relationships.

**Patient and Public Contribution:**

Three public contributors shaped the design of this study by sharing their experiences and views of key issues people who lack adequate social support may experience and how this might impact management of their health. They noted the importance of speaking to a range of people to understand social relationship needs of people living with MLTC, as experiences will vary. Two public contributors reviewed and helped refine the interview topic guide and commented on the importance of tone when conducting interviews, to encourage participants to share their experiences. One public contributor supported analysis of the transcripts through coding, discussion, and manuscript review. They highlighted the abandonment expressed by participants and how participants appeared to want to be seen as independent despite wanting and needing more support.

**Trial Registration:**

None.

## 1 Introduction

Approximately 50% of adults aged ≥ 40 years globally are living with two or more long‐term conditions, commonly referred to as multiple long‐term conditions (MLTC) [1, 2]. People living with MLTC have poorer quality of life, functional decline, greater healthcare needs and higher risk of mortality [3, 4, 5]. Social relationships, defined as the connections people have with others including partners, friends, family members, community organisations, and to a lesser extent health professionals [6], play an important role in risk and outcomes of MLTC [5, 7]; Positive social relationships can reduce adverse outcomes of MLTC through improving health literacy, self‐efficacy, coping strategies and self‐management [8, 9, 10, 11, 12]. In contrast, a lack of positive social relationships can make it more difficult to cope with the burden of managing MLTC and result in worse outcomes, including accumulation of additional conditions and greater disability [5, 13, 14]. Negative social relationships, such as unwanted social support or social support that does not meet an individual's need can be stressful, impact autonomy, and adversely affect health outcomes [15].

Recent research has indicated that people living with MLTC do not feel adequately supported to manage their health [10, 16, 17, 18, 19, 20, 21]. A meta‐synthesis on social needs of people living with MLTC reported a need for stronger community connectedness and improved family assistance and relationships [16]. MLTC may negatively impact social relationships, which could further worsen health outcomes [18, 19]. Existing studies have reported increased reliance on family for emotional and financial support as well as difficulty asking for help, particularly from close relationships [10, 16]. Studies also report on patients living with MLTC having to withdraw from certain social relationships due to the physical limitations and challenges of maintaining social roles whilst managing symptom and care burden (e.g., having multiple treatments or appointments) [18, 19, 20, 21, 22, 23]. Interventions aimed at enhancing social relationships of people living with MLTC could greatly improve care and outcomes. However, a richer understanding of why people withdraw and how MLTC can influence these social relationships could help identify the most effective ways to meet the support needs of people living with MLTC.

The Convoy model of social relations theorises that personal and situational factors (such as MLTC) may prevent people from creating networks of relationships that may be maximally beneficial for self‐management support [23, 24]. This model of social relations further suggests weak ties may be a critical part of adults' convoy of social relationships as they experience life‐changing events such as living with MLTC [24]. Research on social networks of people living with long‐term conditions has described the importance of navigating and negotiating social relationships to identify and maximise resources [10, 12, 22, 23, 25, 26, 27]. This literature identified weak ties as an important source of social support; Weak ties were seen as more reciprocal and allowed people to maintain a sense of independence. The role of weak ties in the context of MLTC has been partly demonstrated for individuals with self‐reported good health [28]. Further research on the different types and roles of social relationships experienced by a broader MTLC population (and how this may vary across groups) would contribute to our understanding of people's needs and expectations for social relationships when living with MLTC.

This study explored experiences of social relationships for adults living with MLTC to improve understanding of their social relationship needs. Specifically, we examined: (a) the role and value of social relationships, and (b) processes and contextual factors (e.g., sociodemographic, disability needs) that influence their social relationships, in the context of MLTC.

## 2 Methodology

A qualitative interview study was conducted. We report our findings in line with the Consolidated Criteria for Reporting Qualitative research (COREQ) checklist and Reflexive Thematic Analysis Reporting (RTARG) guidelines [29, 30]. Ethical approval for this study was obtained from the Health Research Authority (ID: 307102) and the Yorkshire & the Humber‐ Sheffield Research Ethics Committee (ref [22]/YH/0100).

### 2.1 Participant Recruitment

Participants were recruited through local charity organisations and general practice (GP) surgeries in Southern England. For recruitment through local charities, a study poster (detailing the study aims, eligibility criteria [people age ≥ 45 years old and living with more than one long‐term health condition and living at home], and study contact details) was emailed and/or posted to charity members and contacts. We explored experiences of adults aged 45+ years due to the high prevalence and impact of MLTC in midlife and beyond and the need to better understand the impact of MLTC in this population [31, 32, 33]. For recruitment through GP surgeries, local National Institute for Health and Care Research (NIHR) Clinical Research Networks were asked to advertise the study to potential GP surgeries. Interested GP surgeries were then asked to display the study poster at their surgeries. One large GP surgery (covering socioeconomically diverse areas) sent postal invitations (mail packs) to eligible patients. This surgery was asked to identify patients with MLTC and screen against exclusion criteria (resident in a care home, in receipt of palliative care, diagnosed with severe mental health condition, lacking capacity to participate, or any other reason deemed by a health professional as unsuitable to participate). A mail pack including a letter of invitation to take part in the study, a participant information sheet, a consent form and a pre‐paid return envelope) was posted to eligible patients.

All potential participants contacted the research team directly for further information. Purposive selection of participants was used to ensure diversity in terms of age, gender, socioeconomic status, and health conditions, where possible. To achieve this, we monitored characteristics of participants. On observing that the sample mostly represented participants identifying as female and white ethnicity at the earlier stage of recruitment, we recruited through additional organisations working with local communities. We prioritised additional recruitment routes, seeking to engage individuals identifying as male and non‐White ethnicity. This included advertising our study through an organisation supporting unpaid carers and engaging with a community with a high proportion of minoritised ethnic communities. We also advertised through GP surgeries in different areas to recruit participants from different socioeconomic status groups. All participants provided informed consent to be interviewed and audio‐recorded.

### 2.2 Data Generation

Data were collected using semi‐structured one‐to‐one telephone interviews conducted by HH (a health researcher with qualitative research experience). Telephone interviews were selected for their convenience and to meet the preferences of participants who may be less comfortable with virtual interviews via teleconference software. HH phoned participants (and received calls from potential participants) using Microsoft Teams Phone (softphone). An interview guide (Table S1) was developed based on the existing literature, study aims and discussions with the research team (including public contributors). The interview guide was piloted (n = 1) and refined before the first interview and further iterated (through discussions with the research team) as interviews progressed to explore emergent views. Interviews lasted between 14 min and 1 h 34 min (average duration: 54 min). This large range is due to a small proportion (*n* = 3) of interviews being kept short due to participants’ breathlessness and/or caring commitments, while some interviews (*n* = 4) included discussions that were not directly related to our topic. All interviews were recorded and transcribed verbatim by an independent transcription company or Microsoft Teams and checked for accuracy by HH.

### 2.3 Data Analysis

Interview data were analysed using reflexive thematic analysis [30, 34]. Interview transcripts were read repeatedly by HH to ensure familiarity. Some interviews were listened to again to check for errors and facilitate analysis. Initial codes were generated and grouped into possible themes. Initial codes were grouped based on similarity (e.g., low mood, lack of energy, not being able to do what they used to). Codes within groups were reviewed and we considered patterns within the data to generate broader themes (e.g., change in sense of self). AR, a public contributor with lived experience of MLTC and loneliness, read and analysed five of the interviews independently. HH and AR compared and discussed initial codes and themes, with AR acting as a ‘critical friend’ [35]. Proposed themes were discussed and refined with the research team (L.M., M.S., A.R.). Data saturation was deemed as no further substantial themes or codes emerged after the 19th interview and themes were well‐developed and well‐understood [36, 37]. Interview data were conducted and analysed over a period of 19 months (June 2023–January 2025). Themes were developed using all interview data and consistent themes were identified across shorter and longer interviews.

### 2.4 Researcher Positioning

H.H. is a mixed‐methods health researcher concurrently working on a quantitative study exploring bidirectional associations between loneliness, social isolation and MLTC. M.S. is a GP and professor of Primary Care Research and L.M. is an associate professor in psychology, both of whom are experienced in qualitative methods and have conducted research on MLTC. H.H. considered how their previous work and assumptions may have influenced the analysis and interpretation of the data. Discussions with A.R. (a public contributor with lived experience of MLTC and loneliness) from study conception to analysis helped H.H. to consider her positioning, allowing closer engagement with the data.

### 2.5 Patient and Public Contribution

Three public contributors shaped the design of this study by sharing their experiences and views of key issues people who lack adequate social support may experience and how this might impact management of their health. They noted the importance of speaking to a range of people to understand social relationship needs of people living with MLTC, as experiences will vary. Two public contributors reviewed and helped refine the interview topic guide and commented on the importance of tone when conducting interviews, to encourage participants to share their experiences. They identified the need to avoid directly asking about how feelings and thoughts (due to loneliness and social isolation) influence health. They further stressed the importance of sharing some personal information (background/family) about the interviewer to help ‘break the ice’ and encourage them to share, and closing the interview on a positive note or signposting where necessary. One public contributor supported analysis of the transcripts through coding, discussion, and manuscript review. They highlighted the abandonment expressed by participants and how participants appeared to want to be seen as independent despite wanting and needing more support.

## 3 Results

### 3.1 Participant Characteristics

Twenty‐two participants were interviewed. Participants were aged between 47 and 96 years old. All participants were of White British ethnicity and just over half (55%) were female. Half of our participants lived alone, half were living in relatively affluent areas, and just over a third (36%) were living in deprived areas. Participants had a range of physical and mental health conditions. Most participants had contact with others at least once a week, either in or outside their home. Table 1 presents the characteristics of study participants.

**Table 1:** Summary characteristics of participants.

Participant characteristics	*n* (%)
Sex	
Male	10 (45.5)
Female	12 (54.5)
Age	
Range	47−96
Median	67
Ethnicity	
White	22 (100)
Non‐White	0 (0)
Marital status	
Never married	4 (18.2)
Married/cohabitating	10 (45.5)
Separated/divorced	2 (9.1)
Widowed	6 (27.3)
Living alone	
No	11 (50.0)
Yes	11 (50.0)
Employment status	
Not working	2 (9.1)
Working	4 (18.2)
Retired	16 (72.7)
Socioeconomic status (SES) determined by area IMD quintile	
1 (Most deprived)	2 (9.1)
2	6 (27.3)
3	3 (13.6)
4	6 (27.3)
5 (Least deprived)	5 (22.7)
Duration of MLTC	
< 5 years	4 (18.2)
≥ 5 years	18 (81.8)
Degree of disability	
None/low	5 (22.7)
Moderate/high	17 (77.3)
Social networks	
Limited	4 (18.2)
Larger	18 (81.8)

### 3.2 Key Themes

Four themes were developed from the data. The four themes were: (a) there is no single route to meaningful social connection, (b) change in sense of self, (c) a need to be seen and understood, and (d) altered interaction.

#### 3.2.1 There Is No Single Route to Meaningful Social Connection

##### 3.2.1.1 Acquaintances and Support Groups

This theme described the different types of relationships participants reported and the roles these interactions played in their life. Most participants, including those living alone, described having regular interactions with acquaintances. Whilst superficial in nature, these relationships were seen as useful. Participants shared how these interactions provided a distraction from their daily lives and health problems. Many joined social groups and felt they benefitted from these; they described being able to discuss their health with others with similar experiences at these groups, which allowed them not to overburden their families. Some developed friendships with others attending their social groups. However, some participants were less satisfied with existing groups, either due to not meeting like‐minded people with similar interests or not finding groups that met their needs as someone living with MLTC.

When you're actually socially mixing with somebody, you're learning about other people and they ‘re learning about you. The interaction is actually beneficial to your health because it makes you feel so much better.

Woman, 78, lives alone, retired, lower SES, moderate disability

I joined them [social groups] just to get out of the house, to meet people. I've got to meet people, because otherwise, I'm just going to go downhill. It's just that, literally, having somebody to talk to…They're not close friends. I just call them acquaintances, but they're there. We have a bit of a laugh. Sometimes some of them have got similar issues to what you've got, and then you can discuss that without having to feel like you're putting pressure on your family.

Woman, 65, lives alone, retired, lower SES, moderate disability

Because I've got a number of things, it's almost like, well, which one [group] to go to. And the things that I have gone to like a little bit around diabetes, I haven't found that very helpful at all.

Woman, 59, lives with partner, working, high SES, moderate disability

##### 3.2.1.2 Closer Ties

Participants clearly distinguished between acquaintances and stronger social bonds. Some participants talked about having close friends or family members who they felt they could always rely on. These relationships were a source of tangible support, helping them meet their daily health and care needs and/or running errands, and also providing emotional connection.

He [son] comes up about twice a week unless I need him for anything. He will come and take me or do something, you know, whatever it is I want.

Woman, 87, lives alone, retired, higher SES, severe disability

I'm now left with two good friends… they'll do anything for me, you know …they'll be across here in no time at all to do anything for me. [Friend] brought me a meal across.

Man, 72, lives alone, retired, lower SES, moderate disability

When I'm feeling low, or whatever, she's there. I just have to contact her, and she'll either come over, she does live quite a way from me. She'll drop everything to come to me if I'm desperate. So, that's been really helpful. My children, one of them runs around after me, takes me to the hospital appointments.

Woman, 65, lives alone, retired, lower SES, moderate disability

The value of diverse relationships was reported across different sociodemographic [age (< 65 vs. ≥ 65 years), area deprivation and retirement status] groups, duration (< 5 vs. ≥ 5 years) of MLTC, degree of disability, and presence of strong social ties (Table S2). Those who were older, retired or had limited social ties appeared to rely more on acquaintances/social groups to reduce feelings of loneliness or social isolation compared to participants who were younger, working and with larger social networks. Participants who were older or with more severe disability described needing both physical and mental support from closer ties, whereas younger participants with shorter MLTC duration or less disability mostly required emotional support and reassurance (Table S2).

#### 3.2.2 Change in Sense of Self

##### 3.2.2.1 Loss of Self‐Identity/Interests

This theme captured the distress participants reported from no longer being able to do the things they used to do and how this impacted relationships with others. Participants described a change in identity from having health conditions that affected their ability to maintain or form relationships. Many struggled with physical limitations that prevented them from being who they used to be or enjoying their hobbies with others. This affected their confidence and led them to avoid certain social interactions or having strained relationships. In contrast, one participant appreciated this change, commenting that having different health conditions resulted in him becoming more open with closer friends and family, and helped him learn more about his health conditions and potential outcomes.

I used to be quite house proud and so I used to sort of get the house looking nice on the weekend… the house doesn't look as nice and I'm, you know, I don't have people over really, because I'm ashamed of how it looks.

Woman, 47, lives with husband, working, lower SES, moderate disability

I guess the fact that my status has gone, you know I don't have a job because historically there is. the jobs I've had have been quite nice to be able to talk about what I do for a living, all those things. And because I haven't got any of that anymore, my self‐respect has sort of gone if you, if you like. And so to try to reestablish relationships with people feels difficult.

Man, 66, lives alone, retired, lower SES, low disability

The walking that we used to like to do when we were on holiday, I can do some of it, but I can't do as much. I'm unstable unless I'm consciously monitoring myself. So if I feel like, you know, I've got to be careful, I don't lose my balance. I don't trip on something. So when I'm walking, I'm concentrating on walking rather than looking at everything around me where they'll be saying, oh, look at this, look at that. It's a bone of contention between us, we did like to do that [walking] together and now I will attempt to do it, but it's not as pleasurable for me as it once was.

Man, 68, lives with wife, retired, high SES, moderate disability

##### 3.2.2.2 High Mental Load

Participants further described the mental load of having MLTC and how managing the different conditions took up much of their physical and mental energy, leaving little room or interest in social connections. Some participants reported how having a combination of mental and physical health conditions compounded this, making them less motivated to or anxious about interacting with others, sometimes even using their physical health condition as an excuse not to meet with friends.

There are times when two or even all three of them [health conditions] come to the surface at the same time and you know that's difficult…I mean, each of them is competing for space in your mind, you know completely and permanently.

Man, 67, lives with wife, working, high SES, no disability

It's difficult because I can be so taken up with my health at some points that it's, well, it's just enough to get through the day and try and work and stuff. I feel like I'm firefighting, right? Get rid of that symptom or that thing and then something else will pop up.

Woman, 59, lives with partner, working, high SES, moderate disability

I just gradually became more and more of a hermit because I just didn't have the energy to do anything apart from hold down my job, really…there's days when my physical energy is low and I can't manage to get up and do anything. And then there's other days when my physical energy seems to be okay, but I still can't get up and do anything.

Woman, 47, lives with husband, working, lower SES, moderate disability

I've got nobody here. Um, that I live with, that I can, if you like, discuss it[health] with. I can sort of sit around and it just compounds itself in my head.

Man, 66, lives alone, retired, lower SES, low disability

##### 3.2.2.3 Low Mood, Depression, Nostalgia

Many participants expressed nostalgia about their past relationships, which they linked to low mood and depressive symptoms. Some of these participants found themselves replacing social connection with potentially unhealthy behaviours such as eating and drinking.

I've lost a lot of the strength I used to have…which can take you down the road of depression, kind of, you know, which is very easy when you've got all these different illnesses.

Man, 76, lives alone, retired, high SES, moderate disability

I reflect on the times when I was in relationships and had lots of friends around me, was in employment and you know, had lots of contact with other people and had a quite a, you know, quite a diverse social life. And I do go through periods of regret and like I say, melancholy about not having those things anymore. Um, and I find I'm replacing. I'm replacing those relationships with, uh, just watching TV and things like that…There was a period where I was going out but feeling quite anxious about going, leaving the house…It became easier not to associate with people and the more I was isolated, the more I didn't want to be around people…

Man, 66, lives alone, retired, lower SES, low disability

I often don't have any motivation to do anything. And I mean like things I used to like doing or anything. All I get pleasure from these days is eating, drinking and sleeping.

Woman, 75, lives alone, retired, lower SES, moderate disability

Loss of self‐identity/hobbies, mental stimulation, and low mood due to MLTC were experienced by participants from varying sociodemographic characteristics and with varying duration of MLTC, degree of disability (Table S2). However, participants from more affluent areas, with longer duration of MLTC, moderate to severe disability, and with strong ties reported finding ways to adapt to changes and improving their mood through interacting with others. Some working participants and those with mental health conditions, described high mental load as a barrier to engaging with others at times. In contrast, retired participants and those with existing social ties described engaging with others to help reduce this mental load.

#### 3.2.3 A Need to be Seen and Understood

##### 3.2.3.1 Invisible Illness

This theme captured a discrepancy between participants' reality and how they felt others perceived them. Participants said that others (friends, family) often did not understand how much their health conditions affected them, and instead seemed to think they were exaggerating, being lazy, or finding excuses not to go out or engage in social activities.

People see you and they think you're well, because you haven't got, you know, something around your neck or you haven't got your leg in the cast.

Man, 67, lives with wife, retired, higher SES, moderate disability

Whilst they've kind of they've been told I've got health issues… sometimes they don't absorb that…They see the evidence of their own eyes. and they just sort of forget about the rest.

Woman, 47, lives with husband, working, lower SES, moderate disability

##### 3.2.3.2 Sharing Perspectives

Participants discussed the importance of being able to talk about their health with (and gain advice from) people with similar health conditions and experiences. However, they reported finding it difficult to share their experiences and needs with others (friends, family). Participants described feeling as though people generally did not want or have the time to understand how their health conditions impacted them. Participants reported withdrawing from these relationships due to fear of burdening others or feeling they didn't have much to offer.

You end up trying to explain that you've got health issues and maybe come across people that just don't really have the time to hear you out. And you know, and sort of, well, that sounds like a you problem to me.

Woman, 47, lives with husband, working, lower SES, moderate disability

People generally speaking, when they,. when people say, well, how are you? They don't really want to hear how you are. They expect to say, oh, we're doing OK, you know, fine. You know, that's what they expect.

Man, 68, lives with wife, retired, high SES, moderate disability

[when feeling unwell] I sort of want to stay away from people because I don't want to upset them. I don't always want sympathy.

Woman, 53, lives with partner, working, low SES, low disability

Participants appeared to feel as though they needed to be more independent. There was a sense of distress as participants gave accounts of finding ways to manage their health and care on their own so that they didn't have to rely on others as much, and would instead be seen differently.

I do as much as I can myself. My [family member] was taking me to the hospital for lots of my appointments. Then I overheard [them] saying to somebody, ‘Oh, I take mummy, and I take mum there.’ I thought, Right, I can't be doing this. So, I found that there's a bus at the bottom of my road that can take me straight to the hospital. So, I've been doing that. Rather than get [them] to take me. It just made me feel bad. That was being said about me behind my back.

Woman, 65, lives alone, retired, lower SES, moderate disability

I'm more of a problem if I visit than a useful person. Well, that has been because of my needs so far, you know. So I hope if I can, if I can bear to try and increase my fitness, which I hate the idea of, but I don't have to be sick if I'm going to be of any use to them.

Woman, 75, lives alone, retired, lower SES, moderate disability

[in relation to befriending services] it's all a bit one‐sided in that you're giving out all your information, but, basically, they're anonymous, and that puts a barrier up.

Woman, 78, lives alone, retired, lower SES, moderate disability

Many participants talked about a lack of long‐term support from health and care services and organisations, making participants feel disappointed and abandoned.

It's just that all of these things [health problems] sometimes they get you down because there seems to be no consistent help, if you see what I mean. People do help, the various organisations and things, but there's usually only two or three times they speak to you or whatever, and then you're closed down again, for one reason or another… Once they've done their bit, they wipe their hands of you. That's what it seems to me.

Woman, 78, lives alone, retired, lower SES, moderate disability

The need to be seen and understood did not appear to vary by participants’ sociodemographic characteristics, duration of MLTC, functional impairment status, or whether or not they had social ties (Table S2). All groups also reported benefiting from sharing experiences with others. However, participants with mental health conditions and those in less affluent areas also described withdrawing or difficulty sharing their experiences with others they felt could not understand.

#### 3.2.4 Altered Interaction

##### 3.2.4.1 New Ways to Interact

While many participants expressed the challenges of not always being understood by family and friends, these relationships were important to them. All participants, apart from those with limited social ties, reflected on ways they were able to maintain relationships despite their health limitations (Table S2). Some participants used technology to engage with friends and family when they did not feel able to go out.

What I've done is I utilise the way I work and the type of work I do and like using lunch hours to make calls or texts to friends and things like that. emailing and texting, what has been a big boon to me over the years as it's come in, because the other thing is the more tired or unwell I get, the less I want to talk to people on the phone. So being able to e‐mail or, and now text people text is brilliant for me.

Woman, 59, lives with partner, working, high SES, moderate disability

Things like that [instant messaging] software like that that you can have on your phone or your computer and kind of talk to people from home, that helps quite a lot because you can kind of keep up to date with what's going on in their lives and vice versa, but without needing to get off the sofa.

Woman, 47, lives with husband, working, lower SES, moderate disability

A few participants gave accounts of how their relationships with others changed as a result of their health conditions. Some enjoyed visits from friends in their home rather than meeting outdoors.

I don't go in the pubs anymore now, you know, I don't. Well, I do miss the company, but I've got my nice house here. And when [friend] comes round, he'll sit down, munch away at my biscuits. Usually if there's an ice cream in the fridge, he'll help himself to that but he sits down with me and he just talks and then when he's, you know, about an hour later [he] decides, oh, the pub must be open, like it's been lovely seeing you. I'll see you again in a couple of days. And I just say to him, right, well enjoy your pint and off he goes.

Man, 72, lives alone, retired, lower SES, moderate disability

Some participants talked about how the dynamics of their relationships with their significant others had changed due to the significant other having to care for them, which also resulted in reduced social interaction with their own friends. There was a sense of frustration at lacking control over their lives and needing to depend on others.

Our relationship isn't the same as the normal marriage because she says, ‘hey, I'm your nurse, I'm your carer, I love you and you're my husband. But things have changed. We don't have the same relationship you normally would have in a, in a, in a loving relationship. It's not that she doesn't love me. It's not that I don't love her. And yes, we're that much older now, but your relationships change because of the illnesses’.

Man, 67, lives with wife, retired, higher SES, severe disability

Now one of my neighbours comes in once a fortnight and changes the bed for me and where necessary cleans it, cleans the windows. But there is so much going on in her life anyway, I can't ask her to do any more.

Woman, 80, lives alone, retired, high SES, moderate disability

On my really bad days, she [daughter] comes and helps me get showered and dressed. Even sometimes come back and help me to get undressed if I'm having difficulty. It's quite difficult to ask them to do it, though.

Woman, 65, lives alone, retired, lower SES, moderate disability

## 4 Discussion

This study provides a deeper understanding of experiences of social relationships for adults living with MLTC and processes that influence these social relationships. Four distinct themes were identified, which captured diversity in how people experienced meaningful social connection, the influence of an altered (MLTC) identity on social relationships, and a need to be better understood, acknowledged and supported by friends, family, and healthcare services. Our findings indicate that the physical limitations of MLTC required participants to find new ways of maintaining social connection, recognising the value of acquaintances as well as longstanding relationships. Participants often experienced high mental burden, low physical energy, low mood and depressive symptoms as a result of their MLTC. This affected their sense of self and subsequent frequency and quality of their social relationships, often causing them to withdraw from relationships with family and close friends. Participants felt others did not always understand the impact of MLTC, contributing to further withdrawal from social relationships.

### 4.1 Comparison With Existing Literature

Our findings are consistent with existing studies that have examined experiences of living with MLTC [16, 17, 18, 19, 20, 21, 22, 23, 38]. These previous studies have described the importance of social relationships for managing MLTC as well as the challenge of maintaining social relationships due to loss of self‐identity and changes in social roles resulting from physical limitations of MLTC. Our analysis extends this literature by identifying specific pathways and processes through which MLTC influences social relationships ‐ specifically reasons people may withdraw from social relationships. Low mood, anxiety, depressive symptoms, nostalgia, and high mental burden affected participants sense of self and subsequently their interactions with others. Previous research indicates that depression mediates the negative impact of social support and quality of life for MLTC [39]. However, this study suggests that depression may further contribute to lack of social support. Similarly, our finding of a possible negative influence of nostalgia on social relationships contrasts with quantitative research that found nostalgia increased help‐seeking, though impact may vary by type of nostalgia [40, 41].

Our findings are also in line with previous studies that have investigated the social needs of adults living with MLTC. These studies have reported on experiences of loneliness in adults and the need for stronger relationships to help them cope with their health [16, 17, 18, 19, 20, 21, 22, 23, 42, 43, 44]. Our study improves understanding of these needs by describing people's everyday interactions and understanding what can represent a strong or valuable relationship. In doing so, our study found that meaningful relationships can be experienced through diverse relationships, with interactions with acquaintances also seen as a valuable resource; participants benefited from shared understanding and additional support that may not be available through family or other closer relationships. This importance of weak ties for self‐management on MLTC is consistent with previous studies on populations with long‐term conditions [25, 26, 27] as well as the convoy model of social relationships which posits changes in the ways people connect with others as their personal and situational factors change and an increasing importance of weaker ties as people age [24]. The value of longstanding relationships and the use of technology to help maintain these relationships was also observed, in line with previous studies [10, 45]. Similar to recent findings reporting age differences in establishing and maintaining social relationships during life events, we observed some differences in how adults of differing sociodemographic characteristics, duration of MLTC, and degree of disability experienced social relationships [46].

### 4.2 Implications for Research and Health and Care Services

This study has identified potential processes through which MLTC influences social relationships. Future empirical research should test mediation effects of these factors and consider how interventions could target these to allow people to maintain their social relationships. The role of depression as a mediator of the influence of social relationships on MLTC has been evidenced, but further evidence is needed for the reverse relationship. Further, this study indicates that people may not ask for the support they need because they believe they should be and want to be seen as independent. Additionally, our findings identified contextual (sociodemographic, degree of disability, level of social networks) differences in experiences of social relationship needs. Health and social care professionals could take these findings into account when discussing management plans and support groups with patients. The study has also captured people's experiences of social groups and existing interventions and suggest further efforts to connect people to these forms of relationships could be an effective way to meet the support needs and improve self‐management of MLTC. While many reported benefitting from social groups, others noted that these services currently did not meet their needs as individuals living with MLTC. These findings may be used to help improve services, allowing people to feel better supported.

### 4.3 Strengths and Limitations

A strength of our study is the inclusion of participants across a wide age range and with various mental and physical health conditions, allowing different perspectives to be obtained. However, as most participants were of White ethnicity and a larger proportion were living in more affluent areas, experiences of minoritised ethnic communities and those living in deprived areas are likely not captured here. While efforts (e.g., going into a relevant community group) were made to increase diversity in recruitment, this did not increase research participation from minoritised ethnic communities. It is possible that some people may have been less comfortable with telephone interviews, though there may be other reasons for nonresponse. Face‐to‐face interviews may have yielded greater response and more in‐depth data, though recent research suggests similar findings from telephone and in‐person interviews [47]. Further research may explore additional approaches to increase diversity in recruitment. Further, this study was based on a single region (South England) in the United Kingdom and findings may differ for other areas with different neighbourhood characteristics and provision of services such as social prescribing. A second limitation is that we were not able to explore how experiences of social relationships vary by different combinations of health conditions due to the low numbers of people with similar conditions. It is likely that experiences vary by combinations of conditions, and understanding these differences could help tailor interventions. Similarly, we did not explore how experiences vary for certain subgroups including people with learning disabilities and lesbian, gay, bisexual, transgender and queer or questioning (LGBT) communities, who may experience greater loneliness. A third limitation is the cross‐sectional nature of the study, which required participants to reflect on how their social relationships have changed since having MLTC. A longitudinal approach could provide a richer understanding of people's experiences.

## 5 Conclusion

This qualitative study describes experiences of social relationships for adults living with MLTC and how this may vary by contextual factors (sociodemographic, degree of disability, and level of social networks). This study highlighted that meaningful connection was valued and achieved through diverse relationships, including acquaintances and support groups. These relationships provided shared understanding and additional support that may not be available through family or other closer relationships. Further efforts to connect people to these forms of relationships could help meet the support needs and improve self‐management of MLTC. Participants often experienced high mental burden, nostalgia, low physical energy, low mood, and depressive symptoms as a result of their MLTC that affected their sense of self and subsequently resulted in them withdrawing from social relationships. Interventions that target these factors may be an effective way to support meaningful social connection, though further research is needed to test these relationships.

## Author Contributions

**Hilda Hounkpatin:** conceptualisation, methodology, data curation, investigation, formal analysis, funding acquisition, project administration, writing – original draft, writing – review and editing. **Leanne Morrison:** methodology, formal analysis, supervision, writing – review and editing. **Al Richards:** methodology, validation, formal analysis, funding acquisition, writing – review and editing. **Miriam Santer:** conceptualisation, methodology, formal analysis, supervision, funding acquisition, writing – review and editing.

## Ethics Statement

Ethical approval for this study was obtained from the Health Research Authority (ID: 307102) and the Yorkshire & the Humber‐ Sheffield Research Ethics Committee (ref [22]/YH/0100).

## Consent

Written and oral consent for publication of anonymised quotes from interviews was obtained from all participants.

## Conflicts of Interest

The authors declare no conflicts of interest.

## Supporting information

Table S1 Topic guide.

Table S2: Examples of experiences of social relationships by patient characteristics.

## Data Availability

The data that support the findings of this study are available from the corresponding author upon reasonable request.
