# Supplementary material for: Experiences of Social Relationships for Adults Living With Multiple Long‐Term Conditions: A Qualitative Interview Study
Source: Health Expect. 2025 Sep 10;28(5):e70335. doi: 10.1111/hex.70335 (PMC12422356; doi:10.1111/hex.70335)
Supplement: Supplementary file 1 — Table S1 Topic guide. [file HEX-28-e70335-s002.docx]

Supplementary Table 1. Semi-structured interview topic guide

| 1. **Gaining sense of participants’ social relationships and how they feel about these relationships**  - Can you tell me a bit about your social relationships? By social relationships, I mean social connections (both meaningful and superficial) you may have with someone such as a friend, family member (spouse, siblings), co-worker, neighbour, activity partner.   - *How would you describe your circle of friends and family?* - How do you feel about your social relationships? - Have your social relationships always been like this? - Can you talk me through why this has or hasn’t changed? - How have you kept your relationships going? (if hasn’t changed) OR - Can you attribute this change to anything in particular? (if has changed) |
| --- |
| 1. **Exploring link between social relationships and health**  - Please tell me about your health - *What role do your social relationships have on your health?* - *What role does your health play on your social relationships?* |
