# Supplementary material for: Experiences of Social Relationships for Adults Living With Multiple Long‐Term Conditions: A Qualitative Interview Study
Source: Health Expect. 2025 Sep 10;28(5):e70335. doi: 10.1111/hex.70335 (PMC12422356; doi:10.1111/hex.70335)
Supplement: Supplementary file 2 — Table S2: Examples of experiences of social relationships by patient characteristics. [file HEX-28-e70335-s001.docx]

Supplementary Table 2: Examples of experiences of social relationships by patient characteristics

|  |  | **There is no single route to meaningful connection** | | **Change in sense of self** | | | **A need to be seen and understood** | | **Altered interaction** |
| --- | --- | --- | --- | --- | --- | --- | --- | --- | --- |
|  |  | **Acquaintances, support groups** | **Closer ties** | **Loss of self-identity/ interests** | **High mental load** | **Low mood, depression, nostalgia** | **Invisible illness** | **Sharing perspectives** | **New ways to interact** |
| Age | <65 years | "[on support group]: It's nice to know I can talk to people when I want to, and they can talk to me when they want to. But we don't have to talk all the time" woman, 53, living with partner, working.  “Because I've got a number of things, it's almost like, well, which one [group] to go to. And the things that I have gone to like a little bit around diabetes, I haven't found that very helpful at all.” woman,59, lives with partner, working | "We talk, we help each other, we laugh, we joke, we know each other. We support each other really " woman, 53, living with partner, working | "I used to be quite house proud and so I used to sort of get the house looking nice on the weekend... the house doesn't look as nice and I'm, you know, I don't have people over really, because I'm ashamed of how it looks. " woman, 47, lives with husband, working | “It's difficult because I can be so taken up with my health at some points that it's, well, it's just enough to get through the day and try and work and stuff.. I feel like I'm firefighting, right? Get rid of that symptom or that thing and then something else will pop up.” woman,59, lives with partner, working | "I was chatting to a shop assistant yesterday, I just felt a bit lighter" man, 51, lives with partner, not working | “I don't think I look very unwell either you see, or sound very unwell most of the time. So I think it's difficult for people to understand what they see in me is, is different to what my reality is” woman, 59, lives with partner, working | "when I had a couple of friends and they had their own issues and were quite unwell, that helped me a lot really, because I had someone to talk to about it and they understood" woman, 59, lives with partner, working | “Things like that [instant messaging] software like that that you can have on your phone or your computer and kind of talk to people from home, that helps quite a lot because you can kind of keep up to date with what's going on in their lives and vice versa, but without needing to get off the sofa” woman, 47, lives with husband |
|  | 65+ years | "When you're actually socially mixing with somebody, you're learning about other people and they 're learning about you. The interaction is actually beneficial to your health because it makes you feel so much better" woman, 78, lives alone, retired. "Just a meal with a load of people who are completely different..well, it won't help. It might help some people, but it's having an interest in common that's going to continue to make the friendships" woman, 75, lives alone, retired | “I’m now left with two good friends... they'll do anything for me, you know …they'll be across here in no time at all to do anything for me. [Friend] brought me a meal across.” man, 72, lives alone, retired | “I’ve lost a lot of the strength I used to have…which can take you down the road of depression, kind of, you know, which is very easy when you've got all these different illnesses” man, 76, lives alone , retired | “There are times when two or even all three of them [health conditions] come to the surface at the same time and you know that's difficult…I mean, each of them is competing for space in your mind, you know completely and permanently.” man, 67, lives with wife, working | "There are times where you where you're in pain and you just begin to go down like a depressive spiral." man, 67, lives with wife, working | “if you're in a wheelchair, people are more understanding of the fact that you're not well. When you're walking around with just a stick, you're sort of invisible.” woman, 65, lives alone, retired | " If you sit on your own , all you do is think and sometimes a lot of those thoughts are not good" woman, 87, lives alone, retired | “When you're stuck indoors completely on your own and all you've got is the end of a telephone or your iPad with your FaceTime? And those friends then, when you've got nothing else, are extremely important” woman, 87, lives alone, retired |
| Socioeconomic status (SES) | Lower SES | "[on support group]: It's nice to know I can talk to people when I want to, and they can talk to me when they want to. But we don't have to talk all the time"woman, 53, living with partner, working | On my really bad days, she [daughter] comes and helps me get showered and dressed. Even sometimes come back and help me to get undressed if I'm having difficulty" woman, 65, lives alone, retired | “I often don't have any motivation to do anything. And I mean like things I used to like doing or anything. All I get pleasure from these days is eating, drinking and sleeping.” woman, 75, lives alone, retired | "when things are getting on top of me..it's nice to be able to talk to somebody" woman, 53, living with partner, working | "all I want to do is go to bed and sleep. I get to a point sometimes where I don't even want to be in this world because there's nothing in it for me." woman, 78, lives alone, retired | “Whilst they've kind of they've been told I've got health issues… sometimes they don’t absorb that...They see the evidence of their own eyes ..and they just sort of forget about the rest. " woman, 47, lives with husband. | "I can say things to them [support group] that they'll get that other people won't get," woman, 53, living with partner, working | "My friend, the one who contacts me every day, she's actually paid for all my theatre tickets for me. My financial situation has not been very good." woman, 65, lives alone, retired |
|  | High SES | "Just a meal with a load of people who are completely different..well, it won't help. It might help some people, but it's having an interest in common that's going to continue to make the friendships" woman, 75, lives alone, retired "My regular interaction with other people is my cleaner once a week, my gardener once a week, and my handyman once a week. All of whom I get on with very well. I'd call them friends" man, 62, lives alone, retired. | “If you've got these friends who phone you up you can think about what you've been talking about which is better than sitting thinking and worrying” woman, 87, lives alone, retired | "on the excursions that they organize, I've now got to go for what's called a panoramic one or easy, where you do very little walking, whereas at one time I could do a medium or a strenuous one. You just have to accept that you can't do or it's not wise to do more than you can" woman, 80, lives alone, retired | "When I had a couple of friends and they had their own issues and were quite unwell, that helped me a lot really, because I had someone to talk to about it and they understood" woman, 59, lives with partner, working | “I often don't have any motivation to do anything. And I mean like things I used to like doing or anything. All I get pleasure from these days is eating, drinking and sleeping.” woman, 75, lives alone, retired | “People see you and they think you're well, because you haven't got, you know, something around your neck or you haven't got your leg in the cast” man, 67, lives with wife, retired | "I'm involved in a church and I go when I'm able to. I'm socializing, helping people, encouraging people, and enjoying being encouraged myself" man, 83, lives with wife, retired | “I don't go in the pubs anymore now, you know, I don't. Well, I do miss the company, but I've got my nice house here. And when [friend] comes round, he'll sit down, munch away at my biscuits" man, 72, lives alone, retired |
| Retirement status | Not retired (and currently working) | “interaction and networking and having networks outside of work, social links.. that's an important part of health in keeping you active and keeping you sane basically, which is very different to the closer relationships where they are far more supportive and helpful in terms of particularly reassurance I think -Life isn't so bad” man, 67, lives with wife, working | "We talk, we help each other, we laugh, we joke, we know each other. We support each other really" woman, 53, living with partner, working | I think what's happened with the illnesses is it's made me realise actually I know very little. I don't have as much life experience perhaps as I thought I did and other people can help. And so opening up and having some of those conversations”, man,67, lives with wife, working | “I just gradually became more and more of a hermit because I just didn't have the energy to do anything apart from hold down my job, really" woman, 47, lives with husband, working | "there's other days when my physical energy seems to be OK, but I still can't get up and do anything" woman, 47, lives with husband, working | “you end up trying to explain that you've got health issues and maybe come across people that just don't really have the time to hear you out. And you know, and sort of, 'well, that sounds like a you problem to me' ” woman, 47, lives with husband, working | "when I had a couple of friends and they had their own issues and were quite unwell, that helped me a lot really, because I had someone to talk to about it and they understood stuff woman", 59, lives with partner | “What I've done is I utilise the way I work and the type of work I do and like using lunch hours to make calls or texts to friends and things like that. Emailing and texting, what has been a big boon to me over the years as it's come in, because the other thing is the more tired or unwell I get, the less I want to talk to people on the phone. So being able to e-mail or, and now text people text is brilliant for me.” woman, 59, lives with partner, working |
|  | Retired | My regular interaction with other people is my cleaner once a week, my gardener once a week, and my handyman once a week. All of whom I get on with very well. I'd call them friends man, 62, lives alone, retired | “When I'm feeling low, or whatever, she's there. I just have to contact her, and.. She'll drop everything to come to me if I'm desperate. My children, one of them runs around after me, takes me to the hospital appointments” woman, 65, lives alone, retired | “I guess the fact that my status has gone, you know I don't have a job because historically there is.. the jobs I've had have been quite nice to be able to talk about what I do for a living, all those things. And because I haven't got any of that anymore, my self-respect has sort of gone if you, if you like. And so to try to reestablish relationships with people feels difficult. ” man, 66, lives alone, retired | "It [health] does stay in the forefront of your mind all the time really" woman, 80, lives alone, retired | "It [health issues] makes you very depressed because you're just trying to get on and be normal" man, 67, lives with wife, retired | “if you're in a wheelchair, people are more understanding of the fact that you're not well. When you're walking around with just a stick, you're sort of invisible.” woman, 65, lives alone, retired | “If you've got these friends who phone you up you can think about what you've been talking about which is better than sitting thinking and worrying” woman, 87, lives alone, retired. | "I speak to them every day on the phone. And I've got like a videoconferencing thing on my phone. So I can speak to them and see them", man, 72, lives alone, retired |
| Duration of MLTC | <5 years | “The little boy he sometimes on the way home from his nursery he wants to knock on the door to just say hello. That really does… It's really, really lovely that they can do this for me. It's like they care”, woman, 65, lives alone, retired | "I have friends that help me as and when they can..so I feel like I've got a lot of help and support" woman, 66, lives alone, retired | “I reflect on the times when I was in relationships and had lots of friends around me, was in employment and you know, had lots of contact with other people and had a quite a, you know, quite a diverse social life. And I do go through periods of regret and like I say, melancholy about not having those things anymore. " man, 66, lives alone, retired | "at the beginning when I was on my own, I always used to dwell and think on things, and it makes it worse" woman, 66, lives alone, retired | "They [social groups] stopped me getting depressed, because I've got really low" woman, 65, lives alone, retired | “I've got friends that don't understand my place ..I can't talk about some of my diagnosed.. You can't talk about things people just don't want to listen to”, 59, woman, lives with son, not working | "at the beginning when I was on my own, I always used to dwell and think on things, and it makes it worse" woman, 66, lives alone, retired | "We go to an early-evening disco because nobody can stay up very late these days, and because of my [health] I usually flake out about nine o'clock, anyway" woman, 59, lives with son, not working |
|  | 5+ years | "I have lots of nice people around me who make things better. I don’t have to get miserable often" man, 80, living with wife, retired | "He [son] takes my wheelchair down by three steps at night and up again in the morning. He does all the cooking." woman, 96, lives with son, retired | "I've got a friend down the road who used to come and have lunch, but of course I can't cook it now,  so she only comes for a cup of tea occasionally.. I was quite a good cook" woman, 96, lives with son, retired | " If you sit on your own , all you do is think and sometimes a lot of those thoughts are not good" woman, 87, lives alone, retired | "I feel like my emotions are a little bit flatter now" man, 68, lives with wife, retired | "I just feel like I'm the bottom of the pile when it comes to help" woman, 96, lives with son, retired | "Sometimes some of them have got similar issues to what you've got, and then you can discuss that without having to feel like you're putting pressure on your family" woman, 65, lives alone, retired | "I've got him [friend] to come along to an art group that I go to and I I see him [on not being able to do more active things they enjoyed doing together]" man, 76, lives alone, retired |
| Degree of disability | Low | “I was chatting to a shop assistant yesterday. I just feel a little bit lighter perhaps.. It's sort of a bit more novel”, man, 51, living with partner, not working | "the closer relationships where they are far more supportive and helpful in terms of particularly reassurance I think -Life isn't so bad” man, 67, lives with wife, working, | "often I feel like I'm observing the [old friendship group] interaction rather than connecting with it. I feel sort of one step removed" man, 51, lives with partner, not working | “There are times when two or even all three of them [health conditions] come to the surface at the same time and you know that's difficult…I mean, each of them is competing for space in your mind, you know completely and permanently.” man, 67, lives with wife | "I do go through periods of regret and like I say, melancholy about not having those things anymore." man, 66, lives alone, retired | "Most people they don’t have the time to be willing to listen" man,67, living with wife, retired | "We talk, we help each other, we laugh, we joke, we know each other. We support each other really" woman, 53, living with partner, working | "He's somebody that I met in a forum and he was interested in, I guess, in things I was interested in" man, 68, lives with wife, retired |
|  | Moderate/High | "I'm involved in a church and I go when I'm able to. I'm socializing, helping people, encouraging people, and enjoying being encouraged myself" man, 83, lives with wife, retired | “He [son] comes up about twice a week unless I need him for anything. He will come and take me or do something, you know, whatever it is I want.” woman, 87, lives alone, retired | "Losing my balance has been hard..I've lost just about all my real interests because of it" woman, 59, lives with son, not working | "I have lots of nice people around me who make things better. I don’t have to get miserable often" man, 80, lives with wife, retired | “I’ve lost a lot of the strength I used to have…which can take you down the road of depression, kind of, you know, which is very easy when you've got all these different illnesses” man, 76, lives alone, retired | "They see you, and they think that you're all right, because mine are all hidden. So, they don't realise how difficult it is" woman, 65, lives alone, retired | "when you're actually socially mixing with somebody, you're learning about other people and they're learning about you. The interaction is actually beneficial to your health because it makes you feel so much better " woman, 78, lives alone, retired | “My friend who I go swimming with, will literally drive over here, pick me up, take me swimming, drop me home after because you know, I couldn't manage public transport” woman, 47, lives with husband, working |
| Social networks | Limited ties | “I was chatting to a shop assistant yesterday. I just feel a little bit lighter perhaps.. It's sort of a bit more novel”, man, 51, living with partner, not working | "She [neighbour] has been very good to me in that she does [food shopping] for me because I've got not transport, so I can't get to the supermarket and places."woman, 78, lives alone, retired | [on loss of interest in music]: "My loss of engagement in music is partly to do with not having the right context socially in which music thrives in terms of enjoyment" man, 51, lives with partner, not working. “I've got nobody here. Um, that I live with, that I can, if you like, discuss it[health] with. I can sort of sit around and it just compounds itself in my head” man, 66, lives alone, retired | “I've got nobody here. Um, that I live with, that I can, if you like, discuss it[health] with. I can sort of sit around and it just compounds itself in my head” man, 66, lives alone, retired | "All these things [health issues] sometimes they get, they get you down because there's no consistent help" woman, 78, lives alone, retired | "Some of them [old friends] just sort of pooh-poohed it and said, 'oh, we're all somewhere on the scale or something like that" woman, 75, lives alone, retired | [in relation to health services] “it's all a bit one-sided in that you're giving out all your information, but, basically, they're anonymous, and that puts a barrier up.” woman, 78, lives alone, retired | "To be involved in any kind of activity group, like a low key sports activity, alongside other people would be really good." man, 66, lives alone, retired |
|  | Larger ties | "Another friend of mine I met through gardening, and she helps me as and when she can. I go out as much as I can. At the beginning when I was on my own, I always used to dwell and think on things" woman, 66, lives alone, retired | “My friend who I go swimming with, will literally drive over here, pick me up, take me swimming, drop me home after because you know, I couldn't manage public transport” woman, 47, lives with husband, working | “The walking that we used to like to do when we were on holiday, I can do some of it, but I can't do as much. I'm unstable unless I'm consciously monitoring myself. So if I feel like, you know, I've got to be careful, I don't lose my balance. I don't trip on something. So when I'm walking, I'm concentrating on walking rather than looking at everything around me ” man, 68, lives with wife, retired | "Having somebody to talk to..sometimes some of them have got similar issues to what you've got, and then you can discuss that" woman, 65, lives alone, retired | " If you sit on your own , all you do is think and sometimes a lot of those thoughts are not good" woman, 87, lives alone, retired | “People generally speaking, when people say, well, how are you? They don't really want to hear how you are. They expect to say, oh, we're doing OK, you know, fine. You know, that's what they expect.” man, 68, lives with wife, retired | “we get together and we laugh and we laugh about what's happened to us that week and what's happened at the hospital, what somebody's said, you know, and experiences that we get when we go out in our cars or whatever with other people, it's you have to laugh at it, otherwise you cry. So we do.” man,76, lives alone, retired | "I can jump on to the computer or the iPad and I can have a Zoom conversation, which is what I've been doing." man, 67, lives with wife, working |
